# Exploring Drivers of Work-Related Stress in General Practice Teams as an Example for Small and Medium-Sized Enterprises: Protocol for an Integrated Ethnographic Approach of Social Research Methods

**DOI:** 10.2196/15809

**Published:** 2020-02-11

**Authors:** Esther Rind, Sigrid Emerich, Christine Preiser, Elena Tsarouha, Monika A Rieger, IMPROVEjob-Consortium

**Affiliations:** 1 Institute of Occupational and Social Medicine and Health Services Research University Hospital Tuebingen University of Tuebingen Tuebingen Germany; 2 Centre for Public Health and Health Services Research Core Facility for Health Services Research University Hospital Tuebingen Tuebingen Germany; 3 See Acknowledgments section for more information and for collaborators/group members

**Keywords:** occupational health, work-related stress, small and medium-sized enterprises, general practice teams, ethnography, method triangulation, grounded theory

## Abstract

**Background:**

An increasing shortage of skilled personnel, including medical personnel, has been reported in many postindustrial economies. Persisting and growing trends in absenteeism and incapacity to work due to mental disorders are concerning and have increased political, economic, and scientific interest in better understanding and management of determinants related to the work environment and health.

**Objective:**

This study protocol describes an integrated approach of social research methods to explore determinants of work-related stress in general practice teams as an example for micro, small, and medium-sized enterprises (SMEs).

**Methods:**

The methods applied will allow an in-depth exploration of work practices and experiences relating to psychological well-being in general practice teams. An ethnographic approach will be used to develop an in-depth understanding of the drivers of work-related stress in general practice teams. We will combine participating observation and individual interviews with five to seven general practitioners (GPs), and five to seven focus group discussions with the nonphysician staff (3-4 participants per group) in approximately four GP group practices and one single practice in Germany. Data collection and analysis will follow a grounded theory approach.

**Results:**

The Ethics Committee of the Medical Faculty, University Hospital of Tuebingen, Germany, has approved this study (reference number: 640/2017BO2). Recruitment has commenced with study completion anticipated in mid-2020.

**Conclusions:**

The data from this project will be used in follow-up projects to develop and test an intervention to reduce and prevent work-related stress in GP practices and other SMEs.

**International Registered Report Identifier (IRRID):**

DERR1-10.2196/15809

## Introduction

### Background

This study protocol describes an integrated approach of social research methods to explore work-related stress [1] in the primary care setting as an example for micro, small, and medium-sized enterprises (SMEs). We focus on general practice teams because they are particularly challenged by an increasingly complex, intense, and responsible working environment [2].

An increasing shortage of skilled medical personnel, including general practitioners (GPs) and physician’s assistants, has been reported in many postindustrial economies, such as the United States [3], Canada [4], the United Kingdom [5], and Germany [6]. This development has been linked to demographic change in these societies and other macroeconomic, political, and structural processes (eg, digitalization), which results in changing and challenging working environments that affect the mental health and well-being of employers and employees in all economic branches [7]. Managing economic and human resources are integral components of organizational and institutional development. A shortage of staff, together with a persisting and growing trend in absenteeism [8] and incapacity to work, has initiated political, economic, and scientific interest in better understanding and management of work-related stressors or psychosocial risks [9,10] and resources [11,12].

A European survey with a focus on workplace risks collected responses from approximately 50,000 enterprises, including questions on psychosocial risks and their management [7]. The results show that the proportion of establishments having an action plan to prevent work-related stress ranges from 60% in the United Kingdom to 8% in the Czech Republic (Germany: 20%) [13]. Compared with larger companies, SMEs with fewer than 250 employees have tighter financial and human resources; hence, health and safety can be of low priority, particularly affecting issues concerned with mental health [14]. However, enterprises in the nonfinancial business economy (eg, manufacturing, construction) account for 99.8% (Germany: 99.5%) of all enterprises across all European Union (EU) countries, including Norway and Switzerland, and 66.3% (Germany: 62.9%) of total employment [15], emphasizing the pivotal importance of ensuring health and safety for personnel working in smaller businesses.

Primary care practices are established microenterprises that have to be maintained and developed to be economically viable. Usually organized as single or group practices with independent practice owners, these microenterprises face increasing financial competition from new emerging health business models, such as large group practices or medical care centers in which a growing number of physicians are no longer self-employed [16,17]. Similar to other SMEs [18,19], many GPs close to retirement have difficulties finding successors, which has been related to a variety of factors, including relatively little recognition for GPs compared with specialists and increasingly unmanageable workloads [20]. This can jeopardize the existence of the entire business, which is concerning because the provision of regional health care is put at risk, with the availability of primary care in rural areas particularly affected [21,22].

### Theoretical and Legal Frameworks Concerning Work-Related Stress

Although the proportion of physically strenuous work has been declining in many economically developed countries, growing job insecurity accompanied by high demands on employees’ flexibility and mobility have resulted in a shift from a hazardous physical environment to a more challenging and stressful psychosocial working environment [23]. Several relevant theoretical models have explained the development of work-related stress, such as the job demand-control model [24], the job demand-control-support model [25], the effort-reward-imbalance model [26], and the concept of organizational justice [27]. These models relate potentially harmful (eg, high workload and low scope of decision making) and beneficial working conditions (eg, social support and recognition, financial rewards, job security, organizational justice, or good leadership quality) to both physical and mental health (eg, cardiovascular diseases, depression) and health-related behaviors (eg, smoking and drinking) [7,9,28]. Therefore, these models play an important role in the development of concepts improving occupational health because they allow for the evaluation of measures preventing work-related stress.

From a legal perspective, the EU Agency for Safety and Health at Work provides guidelines for the improvement of working conditions implemented in European Health and Safety legislation based on Article 153 of the Lisbon Treaty [10,29]. Existing recommendations and guidelines concerning psychosocial factors in the working environment address both potentially harmful and protective dimensions [10,30-33]:

1. Work content (eg, job autonomy, completeness of tasks, variability of tasks, required qualification);
2. Work organization (eg, working time arrangements, possibility or need for communication or cooperation and delegation, working procedures, interruptions during specific tasks);
3. Social relations (eg, relationships between subordinates and leading personnel, hierarchies, leadership and managerial abilities);
4. Working environment (eg, workplace design and equipment) or working conditions (eg, noise, lighting); and
5. New forms of work (eg, increasing mobility, flexible and temporary working arrangements, telework, decreasing differentiation between work and leisure).

### Work-Related Stress in Primary Care Practices

Higher levels of work-related stress have been reported in the health care setting compared with other economic sectors. Health care staff are exposed to a variety of well-established predictors of chronic stress, including high expectations in the workplace accompanied by insufficient resources (eg, time and personnel) and a lack of monetary, social, and work-related recognition [11,34]. A comparative study investigating work stress in primary care physicians across three different health care systems showed that the highest levels of physician work stress—due to effort and lack of rewards—were reported by German GPs, followed by physicians in the United States and the United Kingdom [35]. The study also highlighted the importance of work-related rewards (salary, career opportunities, and recognition) compared with other factors promoting or preventing work-related stress. Further research from England [36] and Germany [37,38] demonstrated a relatively high prevalence of psychological distress, perceived chronic stress, and burnout among workers in general practices compared with the general population.

Recent studies investigating psychosocial risks or work-related stressors [9,10] in the primary care setting have included specifically the profession of physician’s assistants. A recent systematic literature review highlighting career and job satisfaction in relation to burnout in physician’s assistants summarized several work-related factors unique to the profession, including the practice setting, team dynamics, and career flexibility [39]. The authors concluded that there is a lack of high-quality studies investigating occupational well-being in health care teams. The number of studies addressing the primary care environment was relatively low (7 of 37), highlighting the lack of research in this area. However, we have identified studies from Germany that provide further evidence for the relationship between psychosocial working conditions and the well-being of physician’s assistants [40-44].

Most studies to date have applied either quantitative (eg, cross-sectional research) or qualitative approaches (eg, interviews or focus group discussions). Participatory observation, an ethnographic approach, has also been applied in the health care setting to, for example, examine interprofessional communication within a clinical setting [45] or research complex interactions between doctors and nurses working in intensive care [46]. We identified very few studies in a medical setting in which ethnographic and other qualitative methods were applied in combination [46-49] to achieve a deeper understanding of aspects and circumstances related to occupational health. Furthermore, none of these were conducted in the general practice setting including both doctors and physician’s assistants, although the majority of patients (>65% in Germany and >95% the Netherlands) are treated by their GP before they see a specialist [50]. Therefore, a better understanding of occupational health and safety of health care personnel outside the hospital setting is of importance to mitigate and manage health system challenges at the local level (eg, dealing with multimorbidity, chronic disease, geriatric conditions, obesity, tobacco and alcohol consumption, provision of a family health strategy and prevention programs), which are key tasks of personnel working in the primary care setting.

### Aims and Research Questions

This protocol is for a study that is part of the research collaboration IMPROVE*job* funded by the German Federal Ministry of Education and Research (BMBF 01GL1751 A, B, C, and 01GL1851D) [51,52]. Researchers from four universities and six disciplines—occupational medicine, primary care, psychosomatic medicine, operations research, health promotion, and epidemiology—will collaborate within four consecutive work packages (see Multimedia Appendix 1). Within the research collaboration, we aim to develop a deeper understanding of factors related to the development and occurrence of work-related stress in primary care teams.

The focus of this protocol is related exclusively to the integrated qualitative approach that will be applied in work package 1. Modifiable, setting-specific factors considered relevant in this context are lack of leadership (eg, inexplicit description of responsibilities, poor prioritization), inefficient work processes (eg, long waiting times for patients, high frequency of interruptions), lack of communication (eg, within the practice team or with patients), insufficient implementation of occupational health and safety measures, and lack of occupational health promotion. The following questions will guide the analysis:

1. How is work organized within GP practice teams?
2. Which work-related resources and stressors are specific to the primary care setting?

Within the research collaboration IMPROVE*job,* the findings will inform the development of a participatory intervention for the prevention of work-related stress within primary care teams (work package 2). Applying a cluster randomized controlled trial, the effect of the intervention will be assessed (work package 3) measuring the primary outcome “job satisfaction” and several secondary outcomes before and after the implementation of the intervention comparing possible changes between the intervention and the control group [53]. Finally, options for the transfer of the results into other medical practices and SMEs of other economic branches will be evaluated (work package 4).

## Methods

### Qualitative Methods

For the last three decades, qualitative methods have become an integral part of health-related and health services research. They provide tools for a comprehensive understanding of complex environmental, social, and cultural relationships within a particular setting. Ethnographic research design has been established in other disciplines but has yet found little application in research related to occupational health and safety. The study design conforms to the consolidated criteria for reporting qualitative research (COREQ) [54].

### Study Design: Ethnography and Grounded Theory

An ethnographic technique will be used, including participatory observation accompanied by interviews with the practice owners and focus group discussions with the physician’s assistants. Observation will be guided by a theoretical framework developed through a transdisciplinary process [55,56] by the IMPROVE*job*-Consortium based on occupational health and safety guidelines [10,30-33], while also considering life course-specific aspects relevant in the working environment (eg, professional training, pregnancy, dealing with illness and/or care, retirement). We will accompany different GP practice teams over a sustained period. This will allow the mapping of everyday work practices in relation to stressors [9,10] and resources [11,12] as they occur. Based on the observations, we will be able to capture aspects which the participants themselves are either not aware of or are unlikely to disclose in an interview situation, such as different forms of verbal and nonverbal communication (eg, gestures or impromptu responses to routine or nonroutine situations), as well as the dependency between work processes and work environment.

To further enrich the observational data, we will conduct a variety of interviews with the practice teams. Because we will experience teamwork and collaboration between the entire practice staff through observation, we decided to discuss additionally occurring aspects and themes separately with doctors and physician’s assistants acknowledging their different roles. This will create an atmosphere as unaffected as possible by structures of hierarchy and dependency. These qualitative interviews will be performed either as single interviews used mainly to collect the practice owners’ individual stories about everyday working life [57], or as focus group discussions [58] to capture the physician’s assistants perspective on how work is organized within the team and which work-related stressors [9,10] and resources [11,12] affect their daily routines [58].

The analysis of all data will follow a grounded theory approach, which is suitable for less-studied and complex research questions comprising the construction, modification, and evaluation of knowledge created through the reciprocal relationship between researchers and participants [59]. Grounded theory provides a systematic method to address initially unstructured data from different collection methods. Data collection and analysis are carried out alternatingly, allowing for the continuous development of emerging codes and themes until no new conceptual insights occur, and theoretical saturation is reached [59,60]. We will apply a triangulation of methods [60] (observation, individual interviews, and focus group discussions) to compare different perspectives (researcher, GPs, and physician’s assistants). Using these methods in combination, we expect to uncover different and potentially hidden work practices and interactions within the observed practice teams to understand, conceptualize, and evaluate how these relate to work-related resources and stressors relevant to the primary care setting.

### Participatory Approach: Advisory Board and Research Support Group

**Figure 1:**
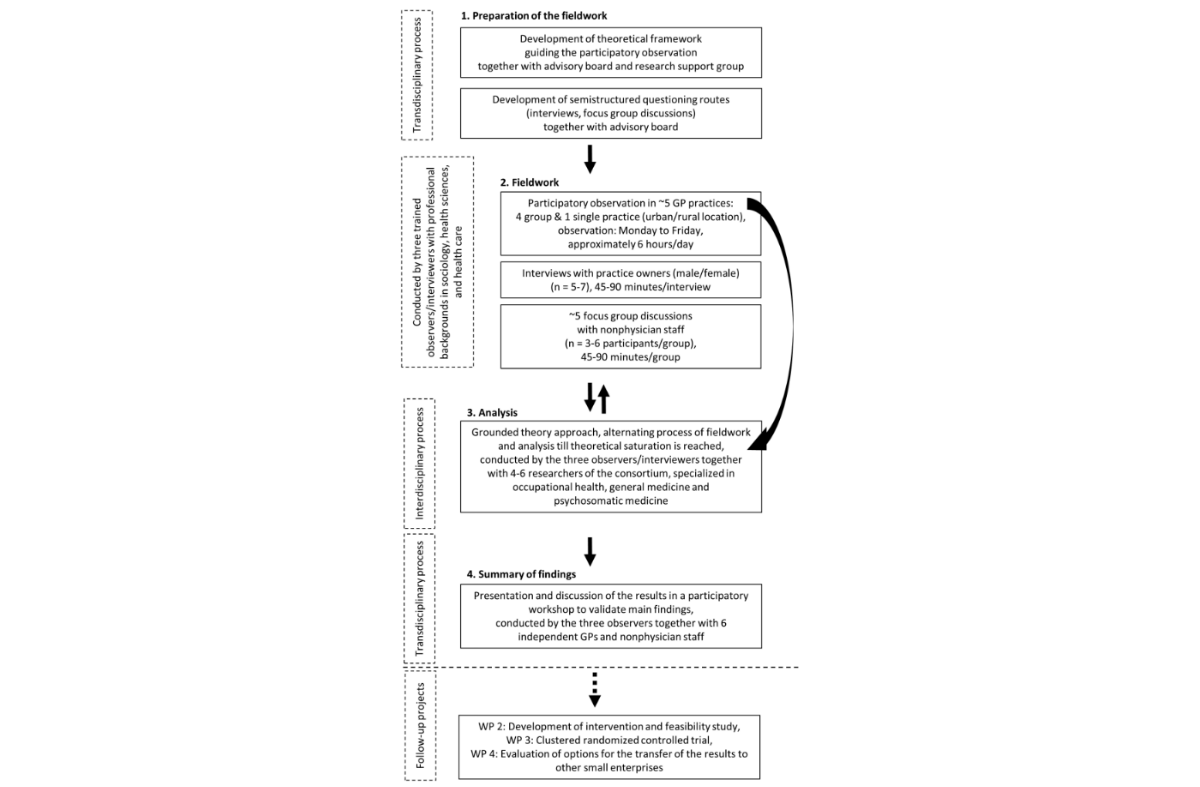
Flowchart of the methods that will be applied in work package 1.

To further ensure scientific rigor, an advisory board will offer guidance and support throughout the study. Members with a scientific and/or professional background in occupational health and safety, work design, or occupational health promotion, as well as scientists from two institutes of general medicine, will provide primarily theoretical and methodological expertise. The research support group, including GP practice staff (doctors and physician’s assistants), will provide advice on the practicalities of applying the ethnographic approach in the general practice setting; furthermore, they will take part in the validation of the emerging codes and themes throughout the analysis to improve the rigor and reliability of the results providing communicative validation [60]. A summary of the methods is displayed in Figure 1.

### Setting and Inclusion Criteria

To capture a variety of primary care settings, we plan to recruit approximately five practices (expected number of practices until theoretical saturation is reached): one single and four group practices in three urban and two rather rural areas, managed by male and/or female practice owners (purposive sampling). Prior research has shown that workload, income, and practice patterns differ frequently to the disadvantage of primary care physicians managing single practices [37] in rural areas [61,62]. Moreover, there is evidence that leadership styles can differ between men and women [63]. Leadership styles have also been related to job satisfaction; for example, transformational leadership has been shown to impact positively on workplace empowerment in the hospital setting, which increased the job satisfaction of nurses and promoted better safety outcomes for both staff and patients [64]. Furthermore, some national [65] and international [66,67] research has shown differences in job satisfaction between male and female GPs, with women being generally more satisfied at work [68]. However, for the perception of chronic stress and burnout, younger, female GPs working part-time have been reported to be more affected than their male colleagues [37,38].

### Recruitment

Access to GP practices is planned through selective sampling via the network of GPs of the Institute for General Medicine, University Hospital Essen (IFAM, Germany). This network is representative of the primary care setting in Germany [69]. All participating practices in this network are located in urban and rural areas of North-Rhine-Westphalia (largest cities: Cologne, Duesseldorf, Dortmund, Essen), one of the most densely populated districts in Germany (population in 2017: 524 per square kilometer) [70]. Suitable GP practices will be invited via post and contacted by telephone if they agree to participate. During the phone call, practice owners will receive detailed information on the study and a schedule for the participatory observation (one working week: Monday to Friday), and the times for the interviews with the practice owners and the focus group discussions will be decided.

### Preparation of the Field Work

In preparation for the fieldwork, all observers and interviewers will participate in a two-day methods course, which will include theoretical background on grounded theory and practical exercises concerning fieldwork and analysis. Each of the three female observers (with professional backgrounds in sociology, health sciences, and health care) will conduct a two-day trial observation in different GP practices to gain first impressions of the setting, its facilities, and organizational structures. The researchers will also have the opportunity to explore their role in the field and develop a feeling for suitable points of observation where they will attract as little attention as possible. 

### Data Collection and Sample Size

Data collection will take place in approximately five GP practices until theoretical saturation is achieved [71].

#### Participatory Observation

To allowing for comprehensive coverage during opening hours over the course of a workweek (Monday to Friday), each practice will be visited daily and in turn by two observers. According to previous studies [45,72], the observation time per person will be 2 to 4 hours to ensure the quality of the fieldwork. In agreement with the practice owners and the patients involved, the observers will attend as many situations and procedures as possible in areas such as reception, the waiting area, the kitchen, and in functional rooms (eg, laboratory, consultation room). Although the researchers will be in continuous contact with practice staff and patients, their aim is not to intervene actively in acute or sensitive events to avoid the disruption of procedures. If possible and appropriate, field notes will be taken, including the documentation of spontaneously occurring conversations with practice staff.

#### Interviews and Focus Group Discussions

All interviews and focus group discussions will follow up aspects that occurred over the course of the observations and will elaborate on particular situations, experiences, attitudes, and ambiguities. For both the interviews and discussions, we developed a semistructured questioning route including an introduction (short thematic intro, information on recording and data protection) and questions related to work content, work organization, social and working environment, as well as new forms of work [10,30-33]. Using a semistructured topic guide will provide the appropriate flexibility to guide the course of the interview. The researchers can clarify questions or can adapt the focus of the interview to further explore topics and phenomena that reoccur in different general practices. Depending on the availability of the practice staff, all individual interviews with the practice owners (approximately 5-7) and discussions (approximately 5, with 3-6 participants per discussion) are planned to last between 45 and 90 minutes. In agreement with the participants, all interviews and discussions will take place in the GP practices and will be digitally recorded, ensuring accuracy and completeness.

### Data Management and Analysis

Observation protocols, complemented by material including spontaneous informal discussions with primary care staff and field notes, will be written by the participating researchers during or immediately following the fieldwork [73]. The transcription of the interviews and focus group discussions will be carried out by a professional company according to a simplified system whereby transcription is word-for-word, but not phonetic [74]. Quality checks, depersonalization, and pseudonymization of all data sources will be done by the team conducting the fieldwork. To facilitate the linkage and classification of all data sources, the software MAXQDA 2018 [75] will be used to organize the observational protocols, interviews, focus group transcripts, and emerging memos.

Data analysis will apply a grounded theory approach including open, axial, and selective coding as well as constant comparison of all material to develop codes, work out the relationships between the codes, and establish a narrative around selected core themes [76]. The analysis will be conducted by the investigators carrying out the fieldwork supported by an interdisciplinary team of researchers from the IMPROVE*job* collaboration with expertise in general, psychosomatic, and occupational medicine.

### Ethical Considerations

Individual declarations of informed written consent will need to be signed by all participating practice staff; each team member will have the opportunity to ask questions and revoke their participation at any time over the course of the study. Data management and storage will be subject to the EU General Data Protection Regulation. The data will be available only to persons involved in this research. Ethical approval for this study was obtained from the Ethics Committee of the Medical Faculty, University Hospital of Tuebingen, Germany (reference number: 640/2017BO2). This study complies with the Declaration of Helsinki, World Medical Association (1964), last revised October 2013 [77].

During fieldwork, all patients and other visitors will be informed about our study at the patient registration desk of the respective practice where a sign will refer to our study and the observing researchers. As working procedures involving patients are of interest only in terms of how work is organized and communicated within the GP team (eg, doctor-patient relationship), patients will be asked by the GP or physician’s assistant whether they agree to the observers being present in the treatment areas, and the observers will sign a declaration of confidentiality. However, we will neither collect nor analyze any patient-related information because this study focuses exclusively on the collaboration within the practice teams. All data sources will undergo the process of pseudonymization.

## Results

Recruitment has commenced, and study completion is anticipated in mid-2020. On behalf of the IMPROVE*job*-Consortium, the findings of this qualitative study (work package 1) will be disseminated via peer-reviewed publications, conferences, and workshops. The results will inform subsequent subprojects (work package 2-4) of the IMPROVE*job*-project, including the development and evaluation of a participatory intervention for the prevention of work-related stress within primary care teams as an example for other SMEs.

## Discussion

### Principal Findings

This study protocol describes an integrated qualitative approach of social research methods to explore drivers of work-related stress in the primary care setting as an example for SMEs, including microenterprises. Criteria to ensure quality have been established, including persistent observation, member checks, and triangulation [60,78,79]. The triangulation of methods applied in this study will allow an in-depth exploration of work practices and experiences in relation to psychological well-being from different perspectives over a sustained period across a variety of GP practice teams, providing particular insight into values and ideas that are inherently difficult to capture (eg, team spirit, attitudes concerning patient care, workplace atmosphere). This is relevant because there is little research to date that applies a comprehensive approach to the study of work-related stress in SMEs such as GP practices. Reducing and preventing work-related stress is an important dimension in protecting the health and safety of employees as well as reducing illness-related costs.

### Benefits of Transdisciplinary Research

The combination of scientific and practical expertise is a central prerequisite for transdisciplinary cooperation [55,56]. Transdisciplinary research networks thrive when an interdisciplinary academic and a practical consensus results in the integration of research ideas and results throughout the entire research process so that jointly developed knowledge and products can be shared with and used by the target audience. It is expected that the entire IMPROVE*job*-project will have a positive effect on the prevention of work-related stress in not only the primary care setting. Other SMEs might benefit from the results learned from the experiences in the GP practices, which may eventually contribute to the promotion of psychosocial aspects of work-related health in other smaller businesses.

### Limitations

From a practical and operational point of view, the recruitment of GPs for research has been shown to be challenging. This has been related to a variety of factors, including the high workload of GPs, skepticism in the applicability of the results, the feeling of being monitored and judged [80], and the sensitivity of the working environment regarding patient confidentiality and data security [81]. Furthermore, the ethnographic approach is prone to disrupting the usual working environment; researchers engage in participatory observation over a sustained period of time [82], which may cause an additional and unusual form of stress. Conducting this study within the collaborative research network, IMPROVE*job* will provide resources to mitigate or overcome some of the challenges described. Over the course of the entire IMPROVE*job*-project, the interdisciplinary research group will work closely together with the study’s target group of GP practice teams. For this subproject, the practitioners of the research support group will provide practical insight into the primary care setting, which will support the researchers with the familiarization of the setting, the recruitment of participating practices, as well as the organization and execution of the ethnographic fieldwork. The researchers will be invited into different GP practices and accompany the team for a week; therefore, we expect all participants (researchers, GPs, and physician’s assistants) to become familiar with this research approach so, ideally, it will cause as little disruption as possible. 

### Conclusion

It is expected that the entire IMPROVE*job*-project will have a positive effect on the prevention of work-related stress inside and outside the primary care setting. Other SMEs might benefit from the results learned from the experiences in the GP practices, which may eventually contribute to the promotion of psychosocial aspects of occupational health in other small businesses.

## Appendix

Multimedia Appendix 1 Evidence based for teams: aims of the transdisciplinary research consortium IMPROVEjob.

Multimedia Appendix 2 Current and former members of the IMPROVEjob-Consortium.

Multimedia Appendix 3 Review and proof ethics-English.

Multimedia Appendix 4 Review and proof ethics-German.

Multimedia Appendix 5 Review and proof of major funding - English.

Multimedia Appendix 6 Original peer review from the Federal Ministry of Education and Research - German.

Multimedia Appendix 7 Original peer review from the Federal Ministry of Education and Research - English.

## Abbreviations

EU: European Union
GP: general practitioner
IFAM: Insitut für Allgemeinmedizin, Universitätsklinikum Essen (Institute for General Medicine, University Hospital Essen)
SME: small and medium-sized enterprise
