# Supplementary material for: Exploring Drivers of Work-Related Stress in General Practice Teams as an Example for Small and Medium-Sized Enterprises: Protocol for an Integrated Ethnographic Approach of Social Research Methods
Source: JMIR Res Protoc. 2020 Feb 11;9(2):e15809. doi: 10.2196/15809 (PMC7055789; doi:10.2196/15809)
Supplement: Multimedia Appendix 1 [file resprot_v9i2e15809_app1.pdf]

IMPROVE*job*
Evidence-based for teams

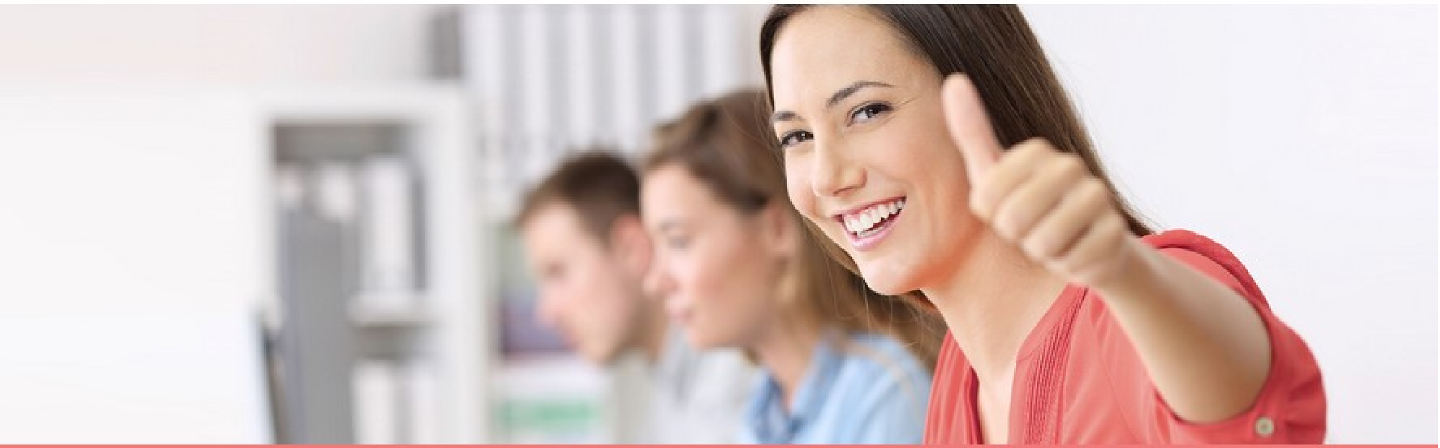

Aims

Using primary care practices as an example for small and medium-sized enterprises (SMEs), IMPROVE*job* aims to develop a multimodal, participatory intervention for the prevention of psychological distress within general practice teams. Furthermore, the effect of the intervention is assessed and options for the transferral into other SMEs are evaluated.

The project is carried out at the universities and university clinics of Tübingen, Bonn, Essen and Bochum in four closely interlinked subprojects (SP):

- SP 1: Analysis of working conditions in primary care practices,
- SP 2: Development of the intervention and feasibility study,
- SP 3: Evaluation of the effectiveness of the intervention (cRCT),
- SP 4: Evaluation of transferral options into other SMEs.

Contact

Coordination IMPROVE*job*-Consortium:
Institute of Occupational and Social Medicine and Health Services Research
University Hospital Tuebingen
Wilhelmstr. 27
D-72074 Tuebingen

+49 7071 29-80154

+49 7071 29-4362

improvejob.projekt@med.uni-tuebingen.de

Federal Ministry of Education and Research
