# Supplementary material for: Exploring Drivers of Work-Related Stress in General Practice Teams as an Example for Small and Medium-Sized Enterprises: Protocol for an Integrated Ethnographic Approach of Social Research Methods
Source: JMIR Res Protoc. 2020 Feb 11;9(2):e15809. doi: 10.2196/15809 (PMC7055789; doi:10.2196/15809)
Supplement: Multimedia Appendix 2 [file resprot_v9i2e15809_app2.pdf]

<u>IMPROVE*job*-Consortium</u>

Current Institutions and Members:

Institute of Occupational and Social Medicine and Health Services Research, University Hospital Tuebingen, Germany: MA Rieger, E Rind, A Siegel, A Wagner, E Tsarouha

Department of Psychosomatic Medicine and Psychotherapy, Medical Clinic, University Hospital Tuebingen, Germany: F Junne, T Seifried-Dübon, F Stuber, A Herrmann-Werner, S Zipfel

Institute of General Practice and Family Medicine, University Hospital Bonn, Germany: B Weltermann, S Kasten, K Linden, L Degen

Operations Research, Ruhr-University Bochum, Germany: B Werners, M Grot

Institute for Medical Informatics, Biometry and Epidemiology & Center for Clinical Studies, University of Duisburg-Essen, Germany: K-H Jöckel, C Pieper, V Schröder, J-M Bois, A-L Eilerts, M Brinkmann

Former Institutions and Members:

Institute for General Medicine, University Hospital Essen, Germany: C Kersting

Institute of Occupational and Social Medicine and Health Services Research, University Hospital Tuebingen, Germany: S Emerich, S Burgess, M Hippler

Institute of General Practice and Family Medicine, University Hospital Bonn, Germany: A Dreher

Institute for Medical Informatics, Biometry and Epidemiology & Centre for Clinical Trials, University of Duisburg-Essen, Germany: C Ose

Operations Research, Ruhr-University Bochum, Germany: L Koppka (Imhoff), J Block
