# Supplementary material for: Exploring Drivers of Work-Related Stress in General Practice Teams as an Example for Small and Medium-Sized Enterprises: Protocol for an Integrated Ethnographic Approach of Social Research Methods
Source: JMIR Res Protoc. 2020 Feb 11;9(2):e15809. doi: 10.2196/15809 (PMC7055789; doi:10.2196/15809)
Supplement: Multimedia Appendix 3 [file resprot_v9i2e15809_app3.pdf]

EBERHARD KARLS UNIVERSITÄT TÜBINGEN

UNIVERSITÄTS KLINIKUM TÜBINGEN

Ethik-Kommission an der Medizinischen Fakultät der Eberhard-Karls-Universität und am Universitätsklinikum Tübingen

Gartenstr. 47, D-72074 Tübingen, Tel. +49/7071/2977661, +49/7071/295965

INDEPENDENT ETHICS COMMITTEE (IEC) APPROVAL FORM

DETAILS OF INVESTIGATOR/TRIAL

IEC-Project Number: **640/2017BO2** Date of submission: 04.10.2017, 26.06.2018

Investigator (Name, Address):

Sigrid Emerich, B.A.
Institut für Arbeitsmedizin, Sozialmedizin und Versorgungsforschung
Wilhelmstr. 27
72074 Tübingen

Protocol title: **IMPROVEjob - Participatory intervention to improve the psychological well-being in primary care practice teams: a model for structural and behavioural prevention in small and medium-sized enterprises.**
The following documents were reviewed (zuletzt eingereicht): Studienprotokoll Version 3.1 vom 21.06.2018, Persönliches Anschreiben für Praxisinhaber/in, Informationsschreiben und Einverständniserklärung für Praxisinhaber/in, Information und Einverständniserklärung für Einzelinterviews, Information und Einverständniserklärung für Fokusgruppeninterviews, Informationsschild für Patienten/Patientinnen, Schweigepflichterklärung Beobachterinnen, Einverständniserklärung zur Durchführung einer teilnehmenden Beobachtung und Datenerhebung , Rekrutierungsschreiben, Persönliches Anschreiben für Praxisinhaber/in, Praxisaushang

Is the investigator a member of the IEC? ☒ no ☐ yes

IEC DECISION

☒ The study was approved

☐ Conditional approval was granted for the study. /Modifications are required prior to approval.

☐ The study was disapproved: documents may be resubmitted after changes have been made.

The IEC of the University of Tübingen is organized and operates according to ICH-GCP and applicable laws and regulations.

SIGNATURE:

I confirm that the details on this form are correct:

K. Jaschonek	Prof. Dr.med. Karl Jaschonek<br>Chairman, IEC	2019-05-31
Signature of Chairman, IEC	Name	Date

Attachments: Approval letter, List of Committee members and their occupations
