# Supplementary material for: Exploring Drivers of Work-Related Stress in General Practice Teams as an Example for Small and Medium-Sized Enterprises: Protocol for an Integrated Ethnographic Approach of Social Research Methods
Source: JMIR Res Protoc. 2020 Feb 11;9(2):e15809. doi: 10.2196/15809 (PMC7055789; doi:10.2196/15809)
Supplement: Multimedia Appendix 4 [file resprot_v9i2e15809_app4.pdf]

EBERHARD KARLS
UNIVERSITÄT
TÜBINGEN

UNIVERSITÄTS
KLINIKUM
TÜBINGEN

Medizinische Fakultät

Ethik-Kommission

Prof. Dr. med. Dieter Luft
Vorsitzender

Telefon: +49 7071 29-77661
Telefax: +49 7071 29-5965
E-Mail:
ethik.kommission@med.uni-tuebingen.de

Ethik-Kommission an der Medizinischen Fakultät der Eberhard-Karls-Universität
und am Universitätsklinikum Tübingen, Gartenstraße 47, 72074 Tübingen

Frau
Sigrid Emerich, B.A.
Institut für Arbeitsmedizin, Sozialmedizin und
Versorgungsforschung
Wilhelmstr. 27
72074 Tübingen

Duplikat

nachrichtlich:
Frau Prof. Dr. med. Monika Rieger
Frau Dr. Esther Rind

**640/2017BO2**	26.06.2018	04.07.2018
unsere Projekt-Nummer	eingegangen am	Datum

Erhebung von psychischen Belastungen in Hausarztpraxenteams. - Eine Exploration mittels Methoden der empirischen Sozialforschung.
Auflistung der eingereichten Unterlagen und Allgemeine Hinweise, siehe Seite 2

Sehr geehrte Frau Emerich,

die Unterlagen zur o. g. Studie hatten der Ethik-Kommission an der Medizinischen Fakultät der Eberhard-Karls-Universität und am Universitätsklinikum Tübingen bereits zur Beratung vorgelegen. Mit Schreiben vom 12.01.2018 hatte die Kommission Änderungen empfohlen. Mit den jetzt geänderten und ergänzten Unterlagen wurden die Anregungen der Ethik-Kommission weitgehend aufgenommen.

Danach bestehen gegen die Durchführung dieser Studie seitens der Kommission keine Bedenken.

Die Ethik-Kommission gibt folgende Hinweise:

1. Die Zustimmung des Patienten zur Beobachtung der Arbeitsabläufe im Behandlungszimmer ist zu dokumentieren (Studienprotokoll, Seite 9).
2. Der Verweis auf §33 des Datenschutzgesetzes NRW ist im Hinblick auf das Studienzentrum Tübingen durch den Verweis auf die entsprechende Bestimmung des Datenschutzgesetzes des Landes Baden-Württemberg zu ersetzen (Schweigepflichterklärung Beobachterinnen, Seite 31).
3. Der Stichtag zur Rückmeldung ist zu aktualisieren (Rekrutierungsschreiben, Seite 37).

Zudem möchte die Kommission darauf aufmerksam machen, dass die Europäische Datenschutz-Grundverordnung (DS-GVO) zum 25.05.2018 in Kraft trat. Daraus ergeben sich für alle medizinische Forschungsvorhaben, die mit der Verarbeitung personenbezogener Daten einhergehen, veränderte Anforderungen und Informationspflichten gegenüber den Studienteilnehmern. Hinweise zum Umgang mit den im Rahmen einer Studie erhobenen Daten nach Maßgabe der DS-GVO finden Sie auf der Internetseite der Ethik-Kommission

Universitätsklinikum Tübingen
Anstalt des öffentlichen Rechts
Sitz Tübingen
Geissweg 3 • 72076 Tübingen
Tel. 07071/29-0
www.medizin.uni-tuebingen.de
Steuer-Nr. 86156/09402
USt.-ID: DE 146 889 674

Aufsichtsrat
Ulrich Steinbach (Vorsitzender)
Vorstand
Prof. Dr. Michael Bamberg (Vorsitzender)
Gabriele Sonntag (Stellv. Vorsitzende)
Prof. Dr. Karl Ulrich Bartz-Schmidt
Prof. Dr. Ingo B. Autenrieth
Klaus Tischler

Baden-Württembergische Bank Stuttgart
BLZ 600 501 01 Konto-Nr. 7477 5037 93
IBAN: DE 41 6005 0101 7477 5037 93
BIC (SWIFT-Code): SOLADEST600
Kreissparkasse Tübingen
BLZ 641 500 20 Konto-Nr. 14 144
IBAN: DE 79 6415 0020 0000 0141 44
BIC (SWIFT-Code): SOLADES1TUB

Im Übrigen wird auf die Ausführungen im Schreiben der Ethik-Kommission vom 12.01.2018 verwiesen.

Für die Durchführung Ihres Studienvorhabens wünschen wir viel Erfolg.

Mit freundlichen Grüßen

Prof. Dr. med. Dieter Luft
Vorsitzender der Ethik-Kommission

ALLGEMEINE HINWEISE:

Mitglieder der Ethik-Kommission: Prof. Dr. med. Henner Giedke - Psychiatrie, Prof. Dr. med. Jürgen Honegger - Neurochirurgie, Prof. Dr. med. Karl Jaschonek - Innere Medizin, Prof. Dr. med. dent. Bernd Koos – Zahnheilkunde, Prof. Dr. med. Holger Lerche - Neurologie, Prof. Dr. med. Dieter Luft - Innere Medizin, Prof. Dr. med. Klaus Mörike - Klinische Pharmakologie, Prof. Dr. med. Christian F. Poets - Kinderheilkunde, Ulrike Röllecke - Laie, Prof. Dr. iur. Dr. h.c. Georg Sandberger - Rechtswissenschaft, Prof. Dr. med. Dr. phil. Urban Wiesing - Medizinische Ethik

Die Ethik-Kommission an der Medizinischen Fakultät der Eberhard-Karls-Universität und am Universitätsklinikum Tübingen verfährt entsprechend den ICH-GCP-Richtlinien, der Deklaration von Helsinki in der jeweils gültigen Fassung sowie den gesetzlichen Bestimmungen. Die Ethik-Kommission ist gemäß § 41a Arzneimittelgesetz, Geschäftszeichen 2017-385-15954, gemäß § 20 Abs. 7 MPG, Aktenzeichen: Z14-A1871-14924/97, gemäß § 92 Strahlenschutzverordnung, Aktenzeichen: Z 2.1.2-22471/2-EK-012-Ber und gemäß § 28g der Röntgenverordnung, Aktenzeichen: Z 2.1.2-22472/2.EK-013/R registriert.

Die berufsethische und berufsrechtliche Beratung gemäß §15 Abs.1 Berufsordnung für Ärzte in Baden-Württemberg ist für 3 Jahre ab Ausstellungsdatum gültig.
Änderungen im Prüfplan und in der Phase der Umsetzung bitten wir der Kommission mitzuteilen; dabei wären wir Ihnen dankbar, wenn Sie geänderte Passagen deutlich kennzeichnen würden.
Unabhängig vom Beratungsergebnis macht die Ethik-Kommission darauf aufmerksam, dass die medizinische, ethische und rechtliche Verantwortung für die Durchführung einer klinischen Prüfung beim Leiter der klinischen Prüfung und auch bei allen an der Prüfung teilnehmenden Ärzten liegt.
Nach Abschluss der Studie bittet die Kommission um einen abschließenden Bericht.

Auflistung der eingereichten Unterlagen

Studienprotokoll Version 3.1 vom 21.06.2018

Persönliches Anschreiben für Praxisinhaber/in

Informationsschreiben und Einverständniserklärung für Praxisinhaber/in

Information und Einverständniserklärung für Einzelinterviews

Information und Einverständniserklärung für Fokusgruppeninterviews

Informationsschild für Patienten/Patientinnen

Schweigepflichterklärung Beobachterinnen

Einverständniserklärung zur Durchführung einer teilnehmenden Beobachtung und Datenerhebung

Rekrutierungsschreiben

Persönliches Anschreiben für Praxisinhaber/in

Praxisaushang
