# Supplementary material for: Exploring Drivers of Work-Related Stress in General Practice Teams as an Example for Small and Medium-Sized Enterprises: Protocol for an Integrated Ethnographic Approach of Social Research Methods
Source: JMIR Res Protoc. 2020 Feb 11;9(2):e15809. doi: 10.2196/15809 (PMC7055789; doi:10.2196/15809)
Supplement: Multimedia Appendix 5 [file resprot_v9i2e15809_app5.pdf]

PROJECT MANAGEMENT AGENCY FOR THE

DLR Project Management Agency

Federal Ministry
of Education
and Research

DLR Project Management Agency
Heinrich-Konen-Straße 1, 53227 Bonn, Germany

DLR Project Management Agency Department Health

Your reference
Your letter of
Our reference M. Ebert

Your correspondent Dr. Ebert

Telephone +49 228 3821- 1739
Telefax +49 228 3821- 1257
E-mail Michael.ebert@dlr.de

To whom it may concern

26.06.2019

Dear Sir or Madam,

This is to confirm that the research consortium **IMPROVE*job*-Consortium** ("Participatory intervention to improve the psychological well-being in primary care practice teams: a model for structural and behavioural prevention in small and medium –sized enterprises (SMEs)") with the following research projects (RP) is funded by the German Federal Ministry of Education and Research:

- **Coordination of the Consortium, Central Project & subproject RP 1:** 01GL1751A ("Working conditions, occupational health and safety, management and communication in family practices and transfer options in SMEs"**, including the study protocol: "**Exploring drivers of work-related psychological stress in general practice teams as an example for small and medium-sized enterprises – a study protocol for an integrated qualitative approach of social research methods"
(PI: Prof. Monika Rieger, University of Tübingen; amount of funding: 983.492€)

- **RP 2:** 01GL1751B ("Evaluation of the Intervention")
(PI: Prof. Birgitta Weltermann, University of Bonn; amount of funding: 315.540€)

- **RP 3:** 01GL1751C ("Workflows and stochastic modelling")
(PI: Prof. Brigitte Werners; University of Bochum; amount of funding: 67.912€)

- **RP 4:** 01GL1851D ("Development of the Intervention and feasibility study)
(PI: Prof. Birgitta Weltermann; University of Bonn; amount of funding: 571.510€)

During application for funding, the scientists' concepts were peer reviewed by the funding body. Please do not hesistate to contact us in case of further questions.

The German Aerospace Center is a member of the Helmholtz Association. DLR is represented by its Executive Board and persons authorised to act on its behalf. For information, please contact the Head of the Legal Department, Linder Hoehe, 51147 Cologne (DLR Headquarters).

Visitor address:
Heinrich-Konen-Straße 5
53227 Bonn, Germany
Telephone +49 228 3821-0
Internet DLR-PT.de
Certified to ISO 9001

DLR Project Management Agency

Sincerely

i.A.

Dr. Michael Ebert

i.A.

Klassen

Diana Klassen
