# Supplementary material for: Exploring Drivers of Work-Related Stress in General Practice Teams as an Example for Small and Medium-Sized Enterprises: Protocol for an Integrated Ethnographic Approach of Social Research Methods
Source: JMIR Res Protoc. 2020 Feb 11;9(2):e15809. doi: 10.2196/15809 (PMC7055789; doi:10.2196/15809)
Supplement: Multimedia Appendix 6 [file resprot_v9i2e15809_app6.pdf]

PROJEKTTRÄGER FÜR DAS

Bundesministerium für Bildung und Forschung

DLR Projektträger, Heinrich-Konen-Straße 1, 53227 Bonn

DLR Projektträger Bereich Gesundheit

Frau
Prof. Dr. Monika Rieger
Universitätsklinikum Tübingen
Institut für Arbeitsmedizin, Sozialmedizin und Versorgungsforschung
Wilhelmstraße 27
72074 Tübingen

Ihre Ansprechpartner:
Wissenschaftliche Mitarbeiter Dr. Sebastian Poschadel
Telefon +49 228 3821-1120
E-Mail sebastian.poschadel@dlr.de

Administrative Mitarbeiterin Diana Klassen
Telefon +49 228 3821-1202
E-Mail Diana.Klassen@dlr.de

Telefax +49 228 3821-1257

Bonn, 07. Februar 2017

Bekanntmachung des BMBF zur „Förderung von Forschungsverbünden zur Gesundheit in der Arbeitswelt“ vom 23.05.2016
Ihr formloser Antrag „Partizipative Intervention zur Verbesserung des psychischen Befindens von Arztpraxisteams: Ein Modell für Verhältnis- und Verhaltensprävention in KMU (PRIMAjob)“ vom 21.09.2016
Hier: Positive Bewertung Ihres Antrags und Einreichung der Formanträge

Sehr geehrte Frau Prof. Rieger,

vielen Dank, dass Sie sich mit einem Antrag auf die Bekanntmachung beworben haben. Ihr formloser Antrag wird positiv bewertet. Er kann in reduziertem Umfang mit **ca. 1,96 Mio. € (inklusive Projektpauschale)** gefördert werden. Wir bitten Sie nun, die Formanträge für Ihren Verbund einzureichen. Hierzu finden Sie in der Anlage die folgenden Unterlagen:

- Antragsbewertung
  Die Bewertung wurde unter Einbeziehung eines interdisziplinär besetzten Begutachtungsgremiums erstellt. Bitte legen Sie mit den Formanträgen eine Stellungnahme zur Umsetzung der folgenden Auflagen und Empfehlungen vor:
  - Die Berücksichtigung der *Leitlinie psychischer Belastungen am Arbeitsplatz* der *Gemeinsamen Deutschen Arbeitsschutzstrategie* (GDA) ist darzulegen bzw. zu gewährleisten.
  - Das Forschungskonzept ist durch eine Orientierung an verschiedenen Lebensphasen zu erweitern.
  - Die Transkriptionskosten in Teilprojekt 1 sind in Kosten pro Stunde anzugeben.
  - Erläuterung der Kosten für die Workshops in den Teilprojekten TP 2 und TP 3.
  - Die Kosten für die Datenorganisation in Teilprojekt 3 sind um 50 Prozent (54.656 €) zu reduzieren.
  - Die Kosten für das Teilprojekt 3 sind darüber hinaus um rd. 156.000 € zu kürzen, da der beantragte Stellenumfang (16,5 und 12 Personenmonate für wissenschaftliche Mitarbeiter/-

Das Deutsche Zentrum für Luft- und Raumfahrt e. V. ist Mitglied der Helmholtz-Gemeinschaft. Vertreter des DLR sind der Vorstand und von ihm ermächtigte Personen. Auskünfte erteilt der Leiter Allgemeine Rechtsangelegenheiten, Linder Höhe, 51147 Köln (Hauptsitz des DLR).

Besucheradresse:
Bonn-Oberkassel
Heinrich-Konen-Str. 5
53227 Bonn
Telefon +49 228 3821-0
Internet www.PT-DLR.de
Zertifiziert nach ISO 9001

innen) auf Basis der Erläuterungen geringer anzusetzen ist.
- Die in Teilprojekt 4 veranschlagten Kosten für dreimal sechs Personenmonate wissenschaftlicher Angestellter/wissenschaftliche Angestellte werden für identische Arbeitsschritte beantragt und sind näher zu erläutern und ggf. zu kürzen.
- Der empfohlene Förderbetrag reduziert sich somit auf rd. 1.96 Mio. €. In diesem Betrag sind weitere Kürzungen für bisher nicht ausreichend erläuterte Personalkosten in Teilprojekt 4 noch nicht enthalten.

- Hinweise und Checkliste für die Erstellung der Formanträge
  Bitte verwenden Sie für die Erstellung der Formanträge das elektronische Online-Antragssystem **easy-Online** (https://foerderportal.bund.de/easyonline). Den direkten Link zu Ihrer Fördermaßnahme werden wir Ihnen gesondert per E-Mail übermitteln. Bitte vergewissern Sie sich, dass die Formanträge alle auf der Checkliste angegebenen Unterlagen enthalten.

Senden Sie bitte als Verbundkoordinatorin alle Formanträge Ihres Verbunds gebündelt an folgende Postadresse:

DLR Projektträger
Gesundheitsforschung
Dr. Sebastian Poschadel
Heinrich-Konen-Straße 1
53227 Bonn

Wenn alle vollständigen Formanträge des Verbundes bis zum **15.03.2017** vorliegen, kann die Laufzeit Ihrer Verbundvorhaben voraussichtlich zum **01.07.2016** beginnen. Sollte der vorgeschlagene Termin nicht eingehalten werden oder die Antragsunterlagen bis zu diesem Zeitpunkt nicht vollständig vorliegen, müssen der Förderbeginn des Verbundes verschoben werden.

Dies ist eine Zwischenmitteilung zum derzeitigen Stand unserer Prüfung. Ein Rechtsanspruch auf Gewährung einer Zuwendung besteht nicht. Bei Ablehnung von Förderanträgen erhalten Sie keinen Ersatz für angefallene Ausgaben.

Bitte leiten Sie dieses Schreiben an die Mitglieder Ihres Verbunds weiter.

Sollten Sie Fragen haben, rufen Sie uns bitte an.

Wir freuen uns auf die weitere Zusammenarbeit mit Ihnen.

i. A. i. A.

Dr. Sebastian Poschadel Diana Klassen

Anlagen
(1) Antragsbewertung
(2) Hinweise und Checkliste für die Erstellung des Formantrags

Anlage 1: Bewertung des formlosen Antrags

Antrags_ID	Akronym	Koordination	Beantragte Fördermittel
**66**	**PRIMAjob**	**Rieger**	**2.168.674 €**

Der Verbund beschäftigt sich mit der Vermeidung psychischer Belastungen in Arztpraxen. Das wissenschaftlich hervorragend besetzte Konsortium widmet sich somit einer für die betriebliche Gesundheitsförderung sehr relevanten beruflichen Gruppe. Der Forschungsansatz ist klar strukturiert und methodisch sehr gut durchdacht. Darüber hinaus werden plausible Transferoptionen aufgezeigt. Zeit-, Meilenstein und Arbeitsplanung überzeugen ebenso wie die Ausführungen zum Datenmanagement.
Vermisst wird im Gesamtkonzept jedoch eine Berücksichtigung verschiedener Lebensphasen. Darüber hinaus fehlt eine erkennbare Orientierung an der *Leitlinie psychischer Belastungen am Arbeitsplatz* der *Gemeinsamen Deutschen Arbeitsschutzstrategie* (GDA). Die beantragten finanziellen Mittel, insbesondere für Personal, werden als zu hoch eingeschätzt. Die Transkriptionskosten sind überdies nicht genau nachvollziehbar. Kosten für Workshops werden in mehreren Teilprojekten (TP 2 und 3), jedoch nicht weiter erläutert. Die Kosten für das Datenmanagement in Teilprojekt 3 sind überhöht, da sie lediglich die Datenorganisation und nicht auch die Datenerhebung umfassen. Die Kosten für wissenschaftliches Personal in Teilprojekt 3 scheinen überhöht und sind zudem teilweise nicht nachvollziehbar. In Teilprojekt 4 werden ohne weitere Erläuterungen dreimal jeweils sechs Personenmonate für identische Arbeitsschritte veranschlagt.

Votum: Der Antrag wird mit Auflagen zur Förderung empfohlen.

Auflagen:

- Die Orientierung an der *Leitlinie psychischer Belastungen am Arbeitsplatz* der *Gemeinsamen Deutschen Arbeitsschutzstrategie* (GDA) ist darzulegen bzw. zu gewährleisten.
- Das Konzept ist durch eine Orientierung an verschiedenen Lebensphasen zu erweitern.
- Die Transkriptionskosten in TP 1 sind in Kosten pro Stunde anzugeben.
- Die Kosten für die Datenorganisation in TP 3 sind um 50 Prozent (54.656 €) zu reduzieren.
- Erläuterung der Kosten für die Workshops in den Teilprojekten TP 2 und TP 3.
- Die Kosten für 16,5 und 12 Personenmonate für wissenschaftliche Mitarbeiter/-innen in TP 3 sind in der Summe zu hoch und werden zudem nicht ausreichend erläutert. Der empfohlene Förderbetrag wird daher um rd. 156.000 € gekürzt.
- Die in Teilprojekt 4 veranschlagten Kosten für dreimal sechs Personenmonate wissenschaftlicher Angestellter/wissenschaftliche Angestellte für identische Arbeitsschritte sind näher zu erläutern und ggf. zu kürzen.
- Der empfohlene Förderbetrag beträgt somit rd. 1.96 Mio. € (inklusive Projektpauschale). In diesem Betrag sind weitere möglich Kürzungen für bisher nicht ausreichend erläuterte Personalkosten in TP 4 noch nicht enthalten.

Anlage 2: Hinweise und Checkliste zur Formantragstellung

Das elektronische Online-Antragssystem **easy-Online** finden Sie unter https://foerderportal.bund.de/easyonline. Den direkten Link zu Ihrer Fördermaßnahme erhalten Sie gesondert per E-Mail.

Bitte beachten Sie bei der Erstellung der Formanträge folgende Dokumente:

- Richtlinien für Zuwendungsanträge auf Ausgabenbasis (AZA) oder
- Richtlinien für Zuwendungsanträge auf Kostenbasis von Unternehmen der gewerblichen Wirtschaft (AZK) und
- Merkblatt Vorkalkulation für Zuwendungen - Kostenbasis - (AZK 4).

Die Dokumente finden Sie im **Formularschrank des BMBF**:

https://foerderportal.bund.de/easy/easy_index.php?auswahl=easy_formulare&formularschrank=bmbf&menue=block

Die folgenden Erläuterungen sollen Ihnen die Formantragstellung erleichtern. Sie ergänzen die oben genannten Richtlinien. Die Lektüre der Richtlinien ist für die Antragstellung unerlässlich.

I. Checkliste zum Formantrag

**Erforderliche Unterlagen:**	**Prüfen: √**
**Druckexemplar** rechtsverbindlich unterschrieben	☐
**Vorhabentitel** (AZA/AZAP/AZK, Seite 1) in **deutscher Sprache**	☐
**Erläuterungen zum Finanzierungsplan** in easy AZA/AZAP/AZK oder als separate Datei in deutscher Sprache	☐
**Vorlage personenbezogener Daten** bei Beantragung von namentlich bereits bekanntem Personal	☐
**Deutsche Vorhabenbeschreibung mit Verwertungsplan** gemäß der Gliederung zu AZA/AZAP/AZK, Seite 9 in den "Richtlinien für Zuwendungsanträge auf Ausgabenbasis" bzw. „Richtlinien für Zuwendungsanträge auf Kostenbasis von Unternehmen der gewerblichen Wirtschaft (AZK)“	☐
**Ressourcenbezogener Arbeitsplan**	☐
**Optionale Unterlagen:**	**Prüfen: √**
**Dienstwegexemplar*** über das zuständige Landesministerium (gilt nur für Hochschulen)	☐
**Bestätigung über den Abschluss der Kooperationsvereinbarung für Verbundprojekte**	☐
**Angebote** (Geräte, Auftragsvergaben)	☐
**Stellungnahme** zur Umsetzung der Auflagen/Empfehlungen aus der Begutachtung	☐
**Finanzierungszusage/n**	☐
**Bonitätsunterlagen** *(evtl. näher ausführen)*	☐
**KMU-Erklärung**	☐

*nicht erforderlich in: Baden-Württemberg, Bayern, Berlin, Brandenburg, Bremen, Hessen, Niedersachsen, Nordrhein-Westfalen, Rheinland-Pfalz. Sonderregelung Saarland: Dienstwegverzicht nur für die Universität des Saarlandes, jedoch nur, wenn der Antrag über die Grundausstattung hinaus keine weitere finanzielle Eigenbeteiligung der Universität vorsieht - also nur bei Vollfinanzierung der zusätzlichen Ausgaben, Sonderregelung Schleswig-Holstein: Dienstwegverzicht bei **Fach**hochschulen.

II. Allgemeine Hinweise

Deutsche Vorhabenbeschreibung

Die deutsche Vorhabenbeschreibung ist Teil Ihres Formantrags und gemäß der Gliederung zu AZA/AZAP/AZK, Seite 9 in den Richtlinien für Zuwendungsanträge auf Ausgaben- bzw. Kostenbasis zu erstellen. Sie stellt eine Zusammenfassung Ihres Forschungsvorhabens dar. Bitte nehmen Sie – in einer für Laien verständlichen Sprache – zu jedem der in der Gliederung aufgeführten Punkte Stellung.

Für Verbundvorhaben muss zusätzlich eine kurze zusammenfassende Vorhabenbeschreibung des gesamten Verbundes in deutscher Sprache beigefügt werden. Aus der Beschreibung muss die arbeitsteilige Zusammenarbeit der Verbundpartner eindeutig hervorgehen. Zudem ist ein gemeinsamer Verwertungsplan für den Verbund vorzulegen. Wenn die Verwertung im Verbund nur gemeinsam erfolgen kann, ist es ausreichend, ausschließlich einen gemeinsamen Verwertungsplan für den Gesamtverbund vorzulegen. In diesem müssen ggf. teilprojektspezifische Verwertungsabsichten gesondert ausgewiesen werden. Andernfalls sind neben einem Verwertungsplan für den Verbund auch Verwertungspläne für jede Zuwendung erforderlich.

Kurzfassung der Vorhabenbeschreibung

Die Kurzfassung der Vorhabenbeschreibung auf AZA/AZAP/AZK, Seite 5 wird im Internet veröffentlicht unter http://www.gesundheitsforschung-bmbf.de/de/Gefoerderte%20Projekte.php. Die Kurzfassung sollte für Laien verständlich das Projekt beschreiben. Verwenden Sie keine Abkürzungen, Aufzählungen oder Fachwörter, sondern ausschließlich geläufige Begriffe. Bitte fügen Sie eine englische Übersetzung dieses Textes bei.

Sollten Sie mit der Veröffentlichung der Kurzfassung nicht einverstanden sein, legen Sie uns bitte einen veröffentlichungsfähigen Text gleichen Umfangs bei.

III. Weitere Hinweise zu Positionen des Finanzgerüsts

Bitte beachten Sie, dass nur Ausgaben/ Kosten zuwendungsfähig sind, die ausschließlich zur Durchführung des geplanten Vorhabens verwendet werden. Unabhängig davon können Hochschulen und Universitätskliniken eine Projektpauschale erhalten. Abweichungen vom empfohlenen Finanzgerüst sind zu begründen.

Personal

Für jede der beantragten Stellen ist in den Erläuterungen eine kurze Aufgabenbeschreibung unter Hinweis auf die geplanten Vorhabenarbeiten zu erstellen. Die erforderliche Qualifikation (Stellenbeschreibung) und die notwendige Personalkapazität müssen nachvollziehbar dargestellt werden. Bei personenbezogenen Berechnungen sind die zugrundeliegenden Daten beizufügen.

Für jede beantragte Hilfskraft-Stelle werden eine Aufgabenbeschreibung und die Berechnungsgrundlage für das vorgesehene Beschäftigungsentgelt benötigt. Bitte geben Sie dazu die Anzahl der Arbeitsstunden und den Stundensatz mit Ausweisung des Arbeitgeberanteils an. Sollten für Sie die Richtlinien der Tarifgemeinschaft deutscher Länder über die Arbeitsbedingungen der wissenschaftlichen und studentischen Hilfskräfte gelten, sind die entsprechenden Vergütungshöchstsätze zu berücksichtigen.

Verbrauchsmaterialien

Bitte erstellen Sie eine kurz begründete, summarische Zusammenstellung der Verbrauchsmaterialien. Aus dieser muss auch die Berechnungsgrundlage der einzelnen Posten

hervorgehen.

Patente

Mittel für die Anmeldung eines Patentes, die während der Laufzeit initiiert wurden, sowie Mittel für die Aufrechterhaltung eines Patents mit entscheidender Bedeutung für das Vorhaben können in der Regel mit bis zu 10.000 Euro gefördert werden. Bei Unternehmen der gewerblichen Wirtschaft gilt dies nur für KMU.
