# Supplementary material for: Exploring Drivers of Work-Related Stress in General Practice Teams as an Example for Small and Medium-Sized Enterprises: Protocol for an Integrated Ethnographic Approach of Social Research Methods
Source: JMIR Res Protoc. 2020 Feb 11;9(2):e15809. doi: 10.2196/15809 (PMC7055789; doi:10.2196/15809)
Supplement: Multimedia Appendix 7 [file resprot_v9i2e15809_app7.pdf]

Federal Ministry of Education and Research (BMBF), Germany
Department of Health
DLR
Dr. Sebastian Poschadel
Tel. +49 228 3821-1120
Email: Sebastian.poschadel@dlr.de
Administration: Diana Klassen
Tel. +49 228 3821-1202
Email: Diana.Klassen@dlr.de
Fax: +49 228 3821-1257

Bonn, 07.02.2017

Prof. Dr. Monika Rieger
University Hospital Tuebingen
Institute of Occupational and Social Medicine
and Health Services Research
Wilhelmstr. 27
72074 Tuebingen
Germany

Announcement of the BMBF for the "Promotion of research collaborations on health in the working world" from 23.05.2016
Your informal application "Participatory intervention to improve the mental well-being of teams working in general practices: a model for the promotion of mental well-being in small and medium sized enterprises (PRIMAjob)" from 21.09.2016

Here: Positive evaluation of your application and submission of the formal applications

Dear Prof. Rieger

Thank you for applying for the competition notice. Your informal application has been evaluated positively. It can be funded at a reduced rate of approximately 1.96 million €. (including project lump sum). We now ask you to fill in the attached forms for your research collaboration. The following documents are attached:

- Evaluation of the application
  The evaluation was carried out by an interdisciplinary research panel. Please submit with the formal applications a statement on the implementation of the following conditions and recommendations:
  - The consideration of the *guideline on mental stress at the workplace of the Joint German Occupational Health and Safety Strategy (GDA)* should be considered and guaranteed.
  - The research concept is to be developed considering different phases of life span.
  - The transcription costs in subproject 1 are to be calculated as costs per hour. Explanation of the costs for the workshops in subprojects SP 2 and SP 3 have to be included.

- The costs for the data management in subproject 3 have to be reduced by 50 percent (54.656 €).
- In addition, the costs for sub-project 3 have reduced by around €156,000, as the number of posts requested (16.5 and 12 person-months for research assistants) should be reduced on the basis of the explanations provided.
- The costs estimated in subproject 4 for (three times six person-months) research associates/scientific have to be explained in more detail and, if necessary, the costs have to be reduced.
- The recommended amount of funding is thus reduced to approximately €1.96 million. This amount includes further reductions for personnel costs in sub-project 4 which have not yet been sufficiently explained.

- Notes and checklist for the preparation of the form applications:
  Please use the electronic online application system for the preparation of formal applications - easy-Online (https://foerderportal.bund.de/easyonline). We will send you the direct link to your funding measure separately by e-mail. Please make sure that the form applications contain all the documents specified on the checklist.

As the coordinator of your research collaboration, please send all the form applications of your association in a bundle to the following

Postal address:

DLR Projektträger
Gesundheitsforschung
Dr. Sebastian Poschadel
Heinrich-Konen-Straße 1
53227 Bonn

If all complete formal applications of the association are submitted by 15.03.2017, the duration of your joint projects is expected to start on 01.07.2016. Should the proposed date of submission is not met or the application documents are not submitted by this date, the start of the funding of the network must be postponed.

This is an interim announcement on the current status of our review. There is no legal claim to the grant. If grant applications are rejected, you will not receive any reimbursement for expenses incurred.

Please forward this letter to the members of your association. If you have any questions, please call us. We are looking forward to further cooperation with you.

Dr. Sebastian Poschadel Diana Klassen

Appendix

(1) Application evaluation
(2) Notes and checklist for the preparation of the formal application

Appendix 1: Evaluation of the informal application

Application_ID	Acronym	Coordiation	Requested Funding
66	PRIMAjob	Rieger	2 168 674 €

The research collaboration is concerned with the prevention of psychological stress in medical practices. The consortium with excellent scientific staff is thus dedicated to a project that is of great importance for the operational health promotion of a very relevant occupational group. The research approach is clearly structured and methodically very well thought out. Furthermore, plausible transfer options are shown. Timing, milestones and work organisation are just as convincing as the explanations of the data management. What is missing in the overall concept, however, is a consideration of different phases of the life span. In addition, there is no recognizable orientation towards the guideline on mental stress at the workplace of the Joint German Occupational Safety and Health Strategy (GDA). The financial resources requested, especially for personnel, are estimated to be too high. Moreover, the transcription costs are not exactly comprehensible. Costs for workshops will be explained in several subprojects (TP 2 and 3), but no further explanations are given. The costs for the data management in subproject 3 are too high, as they only concern data management and do not include data collection. The costs for scientific personnel in subproject 3 seem to be too high and are also partly incomprehensible. In subproject 4, personnel costs are calculated without further explanations.

Vote: The application is recommended for funding under the subsequent conditions.

Conditions:

- Orientation towards the guideline on mental stress at the workplace of the Joint German Occupational Safety and Health Strategy (GDA) must be demonstrated and considered.
- The concept shall be expanded by an orientation towards different phases of the life span.
- The transcription costs in SP 1 are to be stated in costs per hour.
- The costs for data organization in SP 3 are to be reduced by 50 percent (54,656 €).
- Explanation of the costs for the workshops in subprojects SP 2 and SP 3 have to be given.
- The costs for (16.5 and 12 person-months) for research assistants in SP 3 are too high in total and are also not sufficiently explained. The recommended amount of the subsidy is therefore reduced by around €156,000.
- The costs estimated in subproject 4 for the (three times six person-months) scientific employee/scientific assistant has to be explained in more detail and, if necessary, the costs have to be reduced.
- The recommended amount of funding is thus approximately €1.96 million (including project lump sum). In this amount, further possible reductions for previously insufficiently explained personnel costs SP 4 may occur.

Appendix 2: Instructions and checklist for formal submission

The electronic online application system easy-Online can be found at https://foerderportal.bund.de/easyonline. You will receive the direct link to your funding separately by e-mail.

Please take note of the following documents when preparing the form applications:
- Guidelines for Grant Applications on an Expenditure Basis (AZA) or
- Guidelines for Cost-Based Grant Applications from Commercial Entities Economy (AZK) and
- Information sheet preliminary cost calculation for grants (AZK 4).

The documents can be found here:
https://foerderportal.bund.de/easy/easy_index.php?auswahl=easy_formulare&formularschrank=bmbf&menue=block

The following explanations are intended to make it easier for you to submit a formal application. They supplement the above mentioned guidelines. Reading the guidelines is essential for the application.

I. Checklist for the formal application

**Required documents:**	**Check:** ✓
Legally binding signed printed copy	☐
Preliminary title in German [AZA/AZAP/AZK, page 1]	☐
Explanatory notes to the financing plan [in easy AZA/AZAP/AZK] or as a separate file in German	☐
Submission of personal data when requesting personnel	☐
German project description with an utilization/realization plan according to the structure of AZA/AZAP/AZK, page 9 in the "Guidelines for Grant Applications on an Expenditure Basis" or "Guidelines for Grant Applications on an Expenditure Basis by the commercial economy (AZK)".	☐
Resource based work plan	☐
**Optional documents:**	
Official copy* via the responsible state ministry (applies only to universities)	☐
Confirmation of the cooperation agreement for collaborative projects	☐
Offers (equipment, procurement)	☐
Statement on the implementation of the conditions/recommendations from the evaluation	☐
Finacial commitment/s	☐
Creditworthiness documents (please specify if necessary)	☐
Declaration of small and medium sized enterprises	☐

*not required in: Baden-Württemberg, Bavaria, Berlin, Brandenburg, Bremen, Hessen, Lower Saxony, North Rhine-Westphalia, Rhineland-Palatinate. Special regulation Saarland: waiver only for Saarland University, but only if the application does not include any further financial self-participation of the university - i.e. only if the additional expenses are fully financed, special regulation Schleswig-Holstein: waiver for universities of applied sciences.

II. General notes

German project description

The German project description is part of your formal application and is structured as follows [AZA/AZAP/AZK], page 9 in the guidelines for grant applications. It represents a summary of your research project.

Please include - in a format that can be understood by laypersons - the following information on each of the research projects listed above.

For collaborative projects, a short summary project description must also be provided in German. The description must include the division of labor between the partners of the research collaboration. Furthermore, a joint utilization/realization plan for the research collaboration has to be submitted. If utilization/realization can only be carried out jointly, it is sufficient to submit only one joint utilization/realization plan for the entire research collaboration. In this plan, sub-project-specific utilization/realization intentions must be shown separately, if necessary. Otherwise, in addition to a utilization/realization plan for the research collaboration, utilization/realization plans are also required for each grant.

Summary of the project description

The summary of the project description on AZA/AZAP/AZK, page 5 is available on the Internet published under

http://www.gesundheitsforschung-bmbf.de/de/Gefoerderte%20Projekte.php.

The short version of the project description should describe the study in a way that can be understood by laypersons. Do not use abbreviations, enumerations or technical terms, but only common terms. Please add an English translation of this text.

If you do not agree with the publication of the abstract, please send us a copy of a publishable text of equal length.

III. Further notes of the financial framework

Please note that only expenditure/costs used exclusively for the implementation of the planned project are eligible. Universities and university hospitals can receive a lump sum for the project independently of this. Reasons must be given for deviations from the recommended financial framework.

Personnel

For each of the posts requested, the explanatory memorandum should comprise a brief description of the tasks with reference to the planned project work. The required qualification (job description) and the necessary personnel capacity must be displayed. In case of personal calculations, the underlying data has to be attached.

For each requested student post, a job description and the calculation basis for the planned employment is required. Please enter in addition the number of working hours and the hourly rate with indication of the employer's contribution. If the guidelines [Tarifgemeinschaft deutscher Länder] on working conditions for research assistants and student workers apply, the corresponding maximum rates of salary have to be considered.

Equipment

Please provide a brief, reasonable summary of the equipment requested. The summary should also indicate the basis of calculating the individual items.

Patents

Means of applying for a patent initiated during the term of the project and means of maintaining a patent of vital importance to the project may be funded with up to 10,000 Euros. In the case of commercial enterprises, this applies only to small and medium sized enterprises.
